# Treatment of face and scalp solar (actinic) keratosis with daylight‐mediated photodynamic therapy is possible throughout the year in Australia: Evidence from a clinical and meteorological study

**DOI:** 10.1111/ajd.12295

**Published:** 2015-03-31

**Authors:** Lynda Spelman, Diana Rubel, Dedee F Murrell, Jo‐Ann See, Daniel Hewitt, Peter Foley, Robert Salmon, Delphine Kerob, Thierry Pascual, Stephen Shumack, Pablo Fernandez‐Penas

**Affiliations:** ^1^ Specialist Connect Woolloongabba Queensland Australia; ^2^ Woden Dermatology Canberra, ACT Australia; ^3^ Skin and Cancer Foundation Inc. Victoria Australia; ^4^ University of Melbourne Victoria Australia; ^5^ St Vincent's Hospital, Melbourne Victoria Australia; ^6^ Sydney and University of NSW Sydney New South Wales Australia; ^7^ Central Sydney Dermatology Sydney New South Wales Australia; ^8^ Westmead Clinical School Westmead Hospital University of Sydney Sydney New South Wales Australia; ^9^ Skin and Cancer Foundation Sydney New South Wales Australia; ^10^ St George Dermatology and Skin Cancer Centre Sydney New South Wales Australia; ^11^ Illawarra Dermatology and Laser Clinic Wollongong New South Wales Australia; ^12^ Galderma International Paris France; ^13^ Galderma R&D SNC Sophia Antipolis France; ^14^ Probity Medical Research Inc. Waterloo Ontario Canada

**Keywords:** Australia, daylight irradiance, daylight‐mediated photodynamic therapy, meteorological study, methyl aminolaevulinate, solar (actinic) keratosis

## Abstract

**Background/Objectives:**

Solar (actinic) keratosis (AK) is an emergent concern worldwide and is associated with an increased risk of development of non‐melanoma skin cancer, especially squamous cell carcinoma. Daylight‐mediated photodynamic therapy (DL‐PDT) using methyl aminolaevulinate cream has proved to be an effective, nearly painless, and more convenient alternative to conventional PDT for the treatment of AK. In a phase III, randomised, controlled trial performed in Australia, the mean irradiance (light intensity) received by patients during DL‐PDT treatment, assessed via a spectroradiometer, was 305 W/m^2^ (min. 40 to max. 585 W/m^2^) with similar efficacy irrespective of intensity or dose. The objective of the present meteorological study was to assess the suitability of natural daylight to perform DL‐PDT for the treatment of face and scalp AK during different periods of the year and different geographical locations and latitudes across Australia.

**Methods:**

To determine daylight irradiance during a complete year in eight different geographical locations throughout Australia, we used meteorological software (Meteonorm, Meteotest, Bern, Switzerland), and available solar radiation and weather data from 1986–2005.

**Results:**

The average daily irradiance remained within the levels (40–585 W/m^2^) measured during the clinical DL‐PDT study in Australia, throughout the year and in all geographical locations investigated (yearly average from Darwin 548 W/m^2^ to Hobart 366 W/m^2^).

**Conclusions:**

DL‐PDT for the treatment of face and scalp AK in Australia can be performed effectively throughout the entire year as long as weather conditions permit daylight exposure and allow participants to remain under direct light for 2 h.

**Abbreviations**
AK: actinic keratosis
c‐PDT: conventional photodynamic therapy
DL‐PDT: daylight‐mediated photodynamic therapy
MAL: methyl aminolaevulinate cream
MICB: minimum irradiance inducing clinical benefit
PpIX: protoporphyrin IX

## Introduction

Solar (actinic) keratosis (AK) is a concern worldwide with a prevalence of approximately 13% among the Caucasian population in Brazil,^1^ 15% in England,^2^ and up to 60% in Australia.^3^ AK are common skin lesions that appear after long‐term exposure to UV radiation. The presence of AK is associated with an increased risk of developing non‐melanoma skin cancer, especially squamous cell carcinoma.

Conventional photodynamic therapy (c‐PDT) using methyl aminolaevulinate cream (MAL) under occlusion for 3 h before illumination with a red light‐emitting diode lamp is an effective procedure approved for the treatment of non‐melanoma skin cancers such as superficial and nodular basal cell carcinomas, Bowen's disease^4^ and thin, non‐hyperkeratotic AK on the face and scalp.^5^ European studies have shown that daylight‐mediated MAL PDT (DL‐PDT) is an effective, almost painless, and more convenient alternative to c‐PDT for the treatment of AK, especially in large fields of actinic damage which can easily be exposed to daylight.^6^, ^7^, ^8^, ^9^, ^10^ In 2012 an international consensus concluded that exposure to daylight for 2 h following skin preparation and the application of MAL would be appropriate to achieve a similar efficacy to c‐PDT.^11^

More recently, a phase III, multicentre, randomised controlled study performed from March to June 2012 in Australia compared the efficacy and safety of DL‐PDT with that of c‐PDT in 100 patients with mild AK on the face and scalp. The participants in this study were treated only when weather conditions permitted daylight exposure and allowed them to remain under direct light for 2 h, based on investigator's judgement. The results corroborated previous findings from European studies demonstrating that AK can be treated with DL‐PDT as effectively as with c‐PDT, while inducing significantly less treatment‐related pain, fewer treatment‐related adverse events and high patient satisfaction.^12^

Thus far, DL‐PDT studies have been conducted in Europe and Australia during limited periods of the year. Variations in weather conditions and the abundance of daylight in different geographical locations have raised questions over the feasibility of DL‐PDT and the applicability of the recommended treatment on a global scale. A recent study addressing these issues in Europe and Israel showed that the protoporphyrin IX (PpIX) light dose was influenced by geographical location, weather conditions and the time of year.^13^ Therefore, it was considered essential to investigate the feasibility of DL‐PDT in Australia according to the geographical location (latitude), weather conditions and time of the year.

## Methods

The aim of this meteorological study was to determine daylight irradiance (light intensity), based on available solar radiation and weather data using meteorological software, in different locations and times of the year in Australia. These findings were compared to the irradiance measured by investigators during the recent DL‐PDT phase III study performed in the same country.^12^

### Geographical locations

**Figure 1:**
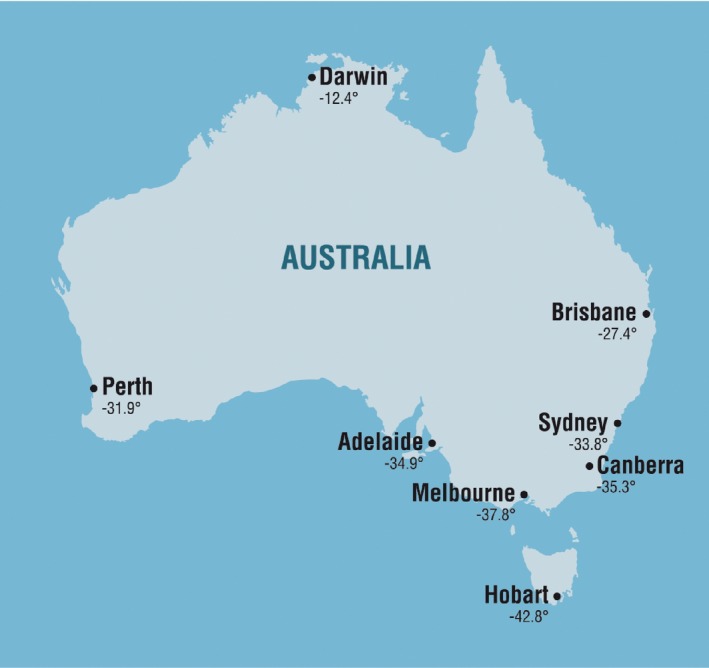
Geographical locations investigated in this study.

A total of eight geographical locations at different latitudes across Australia were investigated in this study, namely Darwin, Brisbane, Perth, Sydney, Adelaide, Canberra, Melbourne and Hobart (Fig. 1).

### Data source

Data in this study were calculated in collaboration with Meteotest (Bern, Switzerland), an expert in worldwide meteorology, environment and information technology.

For this investigation, new atmospheric and global solar radiation data were required. Data combining simulation and existing climatology databases (solar irradiance, weather conditions, and so on) were generated using Meteonorm (http://www.meteonorm.com), the reputable meteorological data software developed by Meteotest. Atmospheric parameters required for modelling irradiance at the ground level (atmospheric turbidity, water vapour and ozone) were also determined using data from the Meteonorm software.^14^ A specific model was developed to study and generate worldwide data for the purpose of DL‐PDT, taking into consideration the PpIX waveband and absorption spectrum. This model was able to use measurements from existing databases as well as interpolate data where measurements were not available, allowing the statistical analysis of a comprehensive dataset in this study.

Furthermore, real irradiance (light intensity) was measured by investigators during the recent DL‐PDT study performed in Australia using light measurement instruments.^12^

## Method

During the Australian DL‐PDT clinical study, the solar irradiance and dose received by participants were measured at clinical sites by all investigators using a spectroradiometer (ILT950, International Light, Peabody, MA, USA) at each site. The instrument measured the spectrum and the amount of energy received at the time of the exposure for DL‐PDT by the participants, from 250 to 1050 nm, expressed in Watts/m^2^ (1 Watt = 1 joule/sec), during the 2‐hour exposure of each subject. A mean irradiance value was obtained and the final dose (mean irradiance × time) was calculated. In this study, no correlation between efficacy and dose or irradiance was observed. Therefore, the range of irradiance received by the participants during this study can be considered as suitable for DL‐PDT.

To compare the clinical study data to the meteorological data we decided to use irradiance values instead of PpIX effective irradiance or dose. Although PpIX effective irradiance (solar irradiance weighted by the PpIX absorption spectrum, that is, the irradiance of the biologically effective part of the solar spectrum) would be a better photobiological measurement, adjusting all study and meteorological data to the PpIX effective irradiance does not add value to the comparison between irradiance levels as the light source (the sun) is the same. Moreover, irradiance is a simpler measurement that can be easily recorded by the standard radiometers that dermatologists could use in their practices. Regarding dose, the reciprocity law states that different combinations of irradiance and exposure time achieving the same dose will have the same effect, but there is no evidence that DL‐PDT (like other photobiological processes) abide by this law. In fact, the transformation of MAL into PpIX requires an undetermined amount of time. If we achieve a fixed dose in less time than that required for the transformation of MAL into PpIX the treatment will be ineffective. Due to these factors, in our article we consider a 2‐h exposure as the amount of time needed to generate a good clinical response, as reported in the Australian phase III study.

Regarding meteorological data, we decided to calculate the mean solar irradiance at ground level (global horizontal irradiance) during office hours (9:00 to 18:00) for an entire year. For practical reasons the meteorological study was limited to office hours as this represents the period of the day when the patients would be visiting the dermatologist's clinic. This is also a period of the day when the influence of the sun's elevation is less likely to range into unwanted lower levels. The values were based on Meteonorm software data for the period 1986–2005. The data presented in the Table 1 are daily averages for each month over an entire year at each location.

**Table 1:** Modelled daily average global radiation for each month Meteonorm data 1986–2005)

Locations	Latitude	Average daily irradiance in W/m^2^												
		Jan	Feb	Mar	Apr	May	Jun	Jul	Aug	Sep	Oct	Nov	Dec	Yearly Average
Darwin	−12.4	507	485	547	538	538	529	554	591	601	580	583	516	548
Brisbane	−27.4	635	575	551	471	358	309	344	396	497	547	584	609	489
Perth	−31.9	737	668	591	410	330	285	292	392	495	591	692	739	518
Sydney	−33.8	610	562	481	410	303	245	267	335	410	488	514	589	434
Adelaide	−34.9	709	669	550	394	273	222	248	338	449	551	641	691	477
Canberra	−35.3	654	612	528	413	295	227	252	329	419	517	599	657	458
Melbourne	−37.8	604	555	485	345	227	193	211	285	387	464	555	598	408
Hobart	−42.8	568	524	400	286	189	154	179	250	347	446	505	552	366

### Assessment criteria

We decided to calculate the minimum irradiance that induced a clinical benefit (MICB) after 2 h of daylight exposure from the DL‐PDT study.

## Results

In the Australian DL‐PDT study the mean irradiance to which participants were exposed during the 2‐h treatment was 305.8 W/m^2^. DL‐PDT sessions during this study were performed between March and May. The irradiance range was from 40 to 585 W/m^2^.^12^ There was no statistically significant correlation between the clinical benefit and solar irradiance and, therefore we considered the MICB to be the lowest average irradiance received during this study, that is, MICB = 40 W/m^2^.

**Figure 2:**
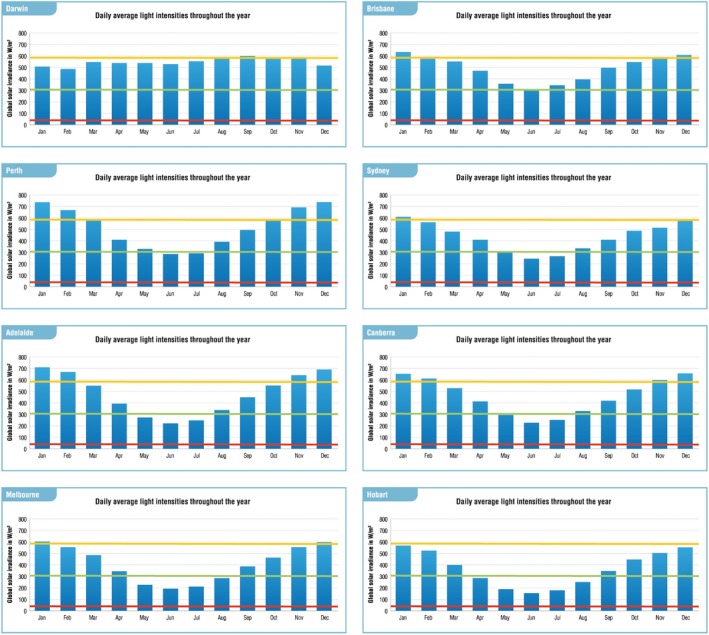
Modelled daily average global radiation for each month (Meteonorm data 1986–2005). Horizontal lines indicate light intensity levels of Australian DL‐PDT study^12^ (red: min. 40 W/m^2^; orange: max. 585 W/m^2^; green: average 305.8 W/m^2^).

Throughout the year, in all Australian geographical study locations, the average daily solar irradiance exceeded the mean irradiance level in the DL‐PDT study (305.8 W/m^2^). Darwin reported the highest yearly average of daily irradiance (548 W/m^2^) followed by Perth (518 W/m^2^) and Brisbane (489 W/m^2^). The location with the lowest yearly average of daily irradiance, although still higher than the mean irradiance in the study, was Hobart (366 W/m^2^) (Table 1) (Fig. 2).

Although average daily sun irradiance below the mean clinical study level (305.8 W/m^2^) was indeed observed, it was still much higher than the MICB (40 W/m^2^). These lower levels were observed in Melbourne during June (193 W/m^2^) and July (211 W/m^2^) as well as in Hobart during June (154 W/m^2^) and July (179 W/m^2^) (Table 1) (Fig. 2).

## Discussion

No relationship between sun irradiance and treatment efficacy was found during the Australian DL‐PDT clinical study.^12^ While irradiance is influenced by geographical location, weather conditions, hour of the day and period of the year,^13^ the reported average irradiance levels in this meteorological study consistently exceeded the MICB received during the recent Australian DL‐PDT study.^12^ Therefore, the findings of this meteorological analysis suggest that weather conditions in Australia throughout the year are appropriate to perform DL‐PDT for the treatment of face and scalp AK. This also suggests that, as the procedure is feasible throughout the year, the main factor to consider before selecting DL‐PDT should be weather conditions rather than season of the year or geographical location. Weather conditions should allow patients to stay outside comfortably for 2 h.

It has previously been demonstrated that treatment effectiveness is reduced at ultra‐low irradiances using artificial daylight sources^15^ but a MICB has not yet been calculated for sunlight. In any case, low irradiance levels in daylight occur under deep shade or during rainy weather, and these conditions were avoided during the DL‐PDT study. Since these weather conditions lead to low levels of light and do not allow patients to stay outside comfortably for the 2 h of required exposure, DL‐PDT should be avoided under these conditions to ensure effective and convenient treatment. Of note, users should bear in mind that this publication presents average data, and that there are variations in real life. In general, sunny and rainy days will have higher and lower irradiance values, respectively, compared to average values presented in this study. Moreover, the irradiance varies throughout the day due to the sun's elevation. Therefore, for practical reasons, the patient should be exposed during office hours to minimise the potential influence of sun's elevation, especially in winter.

Collectively, these meteorological data demonstrate that both light conditions and atmospheric conditions in Australia are suitable throughout the year for the use of DL‐PDT in the treatment of face and scalp AK. During hours of increased risk for exposure to UV radiation, participants receiving treatment should use sunscreen offering adequate sun protection (SPF 30 or higher) that does not contain physical filters such as titanium dioxide, zinc oxide or iron oxide that could interfere with the visible light activation spectrum of PpIX.^16^ Sunscreen should be applied on all sun‐exposed areas including treated areas to protect the skin from exposure to UV radiation and damage.

DL‐PDT is dependent both on the light dose received and the exposure time. Thus, the 2‐h exposure to daylight is essential for the production and activation of PpIX. Shorter exposure to daylight may lead to the insufficient production of PpIX and impair the efficacy of treatment.

Patients are not obliged to remain for 2 h under direct sunlight. When this is uncomfortable (e.g. too hot), they may move intermittently in the shade. However, they should avoid deep shade, where there is a lack of sufficient light, as this may lead to reduced efficacy.

In conclusion, DL‐PDT for the treatment of face and scalp AK can be performed effectively throughout the entire year in Australia as long as weather conditions permit daylight exposure and allow participants to remain comfortably under direct light for 2 h, based on the judgement of the treating physician. Treatment during rainy weather should not be recommended as there are no data to support treatment efficacy under these conditions.
